# Comparative analysis of circulating tumor cells in *prostatic plexus* and peripheral blood of patients undergoing prostatectomy

**DOI:** 10.1186/s13046-025-03397-5

**Published:** 2025-05-13

**Authors:** Gresa Emurlai, Randi M. Pose, Nikhil Kalra, Cornelia Coith, Sandra Lenz, Pierre Tennstedt, Markus Graefen, Stefan Werner, Sabine Riethdorf, Derya Tilki, Simon A. Joosse, Klaus Pantel

**Affiliations:** 1 Department of Tumor Biology, University Medical Center Hamburg-Eppendorf, Martinistr. 52, Hamburg, 20246 Germany; 2 Martini-Clinic Prostate Cancer Center, University Medical Center Hamburg-Eppendorf, Martinistr. 52, Hamburg, 20246 Germany; 3 Department of Urology, University Medical Center Hamburg-Eppendorf, Martinistr. 52, Hamburg, 20246 Germany; 4 Mildred Scheel Cancer Career Center HaTriCS4, University Medical Center Hamburg-Eppendorf, Martinistr. 52, Hamburg, 20246 Germany

## Abstract

**Background:**

The potential influence of radical prostatectomy on tumor cell release into the blood circulation is an under-investigated area.

**Methods:**

One hundred three treatment-naïve patients with early-stage prostate cancer were recruited. Blood from the prostatic venous plexus was analyzed for the local release of tumor cells during radical prostatectomy. Simultaneously, systemic spread was assessed by the presence of circulating tumor cells (CTCs) in peripheral venous blood using both the EpCAM-dependent CellSearch and the size-dependent Parsortix systems in parallel. Tumor cells in the plexus blood and CTCs were detected by epithelial keratin expression and lack of CD45 leukocyte antigen.

**Results:**

Median counts of Keratin + /CD45- cells detected in peripheral blood with the CellSearch and Parsortix systems differed significantly (*p =* 0.0067) with higher sensitivity of the Parsortix System (16 vs 32% positive findings). Even if the results of both assays were combined, the median number of Keratin + /CD45- cells in the prostatic plexus blood was significantly higher than in the peripheral blood (97 vs. 2 per 7.5 ml, respectively, *p* < 0.0001). Keratin + /CD45- cells could be identified in 85% of prostate cancer patients in the prostatic plexus blood and in 42% of patients in peripheral blood during surgery without any significant correlation. Keratin + /CD45- cell clusters were identified in the prostatic plexus in 51.2% of patients but neither these clusters nor single Keratin + /CD45- cells were associated with biochemical relapse during follow-up. The presence (*p =* 0.0094) and number (*p =* 0.0153) of CTCs in the peripheral blood was significantly associated with PSA levels at initial diagnosis. Single-cell genome-wide sequencing by NGS showed copy number alterations (CNAs) in 15 out of 26 index CTCs originating from both the prostatic plexus and peripheral blood compartments.

**Conclusion:**

Combining different CTC enrichment principles increases the CTC detection rate in the peripheral blood of early-stage prostate cancer patients. Our study provides first evidence for a considerable local release of normal and malignant epithelial cells during prostatectomy, which, however, was neither associated with increased CTC detection in the peripheral blood nor with early biochemical recurrence. Longer follow up studies are required to assess whether local tumor cell spread might contribute to clinical outcome in prostate cancer.

**Supplementary Information:**

The online version contains supplementary material available at 10.1186/s13046-025-03397-5.

## Background

For men, prostate cancer is the most common cancer and the second leading cause of cancer-related death [1, 2]. The main risk factors include diet, ionizing radiation, ultraviolet rays, air pollution, and inhaled cigarette smoke, as well as age, family history, and African heritage [3, 4]. Prostate cancer often grows without symptoms for many years and consequently, approximately 15% of the patients are initially diagnosed with advanced stage disease [5]. Treatment options for prostate cancer patients are surgery, radiation-, hormone-, immune-, and chemotherapy, with surgery being the most important intervention [6].

Already at early-stage, tumor cells can disseminate from the primary tumor into the bloodstream [7], spreading to other organs such as the bone to eventually start a metastasis and turning into incurable disease [2, 8]. Early identification of potential tumor cell spread followed by appropriate treatment can significantly improve survival chances. Therefore, to improve our understanding of the processes contributing to tumor cell dissemination as important step in the metastatic cascade is of utmost importance [9–11]. The so-called circulating tumor cells (CTCs) originating from the tumor can be detected in the peripheral blood using various techniques [10, 12, 13].

The question whether invasive interventions such as biopsies or surgery may lead in some instances to an iatrogenic spreading of cancer cells is under-investigated. Previously, we reported data of a pilot study suggesting that transrectal ultrasound-guided needle biopsy might lead to an increase of tumor-associated release of CTCs into the blood circulation and with that to worse progression-free survival in prostate cancer patients [14]. Furthermore, cement augmentation of vertebral metastases has been shown to lead to an increase of CTCs as well [15]. However, to our best knowledge, there is no published information whether and to what extent tumor cells are released during surgical resection of the primary prostate carcinoma. Thus, the present study was aimed to investigate whether surgical intervention by radical prostatectomy can lead to an increased local release of tumor cells into the *prostatic plexus*, and whether this local release results in an increase in CTCs in the peripheral blood circulation as an indicator of systemic tumor cell spread. Therefore, we analyzed, for the first time, the tumor-draining blood from the *prostatic plexus* and peripheral blood in parallel from the same prostate cancer patients undergoing prostatectomy, for the presence of tumor cells.

## Material and methods

### Patients

In total, 103 patients with histologically proven prostate cancer treated at the Martini Clinic Center Hamburg were enrolled between May 2020 and November 2021. This study was approved by the local ethics committee (ethics review board Aerztekammer Hamburg, approval number PV5392) and all patients gave written informed consent. After approximately 1 h into surgery, 15 ml of blood originating from the *prostatic plexus* was collected in ethylenediaminetetraacetic acid (EDTA) containing blood collection tubes (Sarstedt, Nürnbrecht, Germany), as well as 15 ml of peripheral blood at the same time and another 15 ml of peripheral blood directly (up to 2 h) after surgery.

### Peripheral blood processing

For CTC analysis, 7.5 ml of peripheral blood was transferred into CellSave tubes (Menarini Silicon Biosystems, cat.no. CS0018), processed by the Celltracks Autoprep System within 72 h after blood collection using the CELLSEARCH® Circulating Tumor Cell Kit (Menarini Silicon Biosystems) according to the manufacturer’s instructions. Enriched cell fractions were analyzed using the Celltracks Analyzer II. Suspicious cells were presented in an image gallery and evaluated for CTCs by an experienced researcher in blinded fashion. CTCs were identified as, round or oval cells with a diameter of at least 4 µm positive for keratin and DAPI, but negative for CD45. In parallel, another 7.5 ml of peripheral blood was analyzed within 3 h of collection or transferred immediately to TransFix Tubes (Cytomark, cat.no. TVT-09–50) and processed within 72 h with the Parsortix PC1 System (Angle, Guildford, UKE) using capturing cassettes with a size-restricted gap of 6.5 µm and a pressure of 50 mbar according to the manufacturer’s instructions as before [16]. Both blood tubes were processed with the two CTC enrichment techniques in parallel on the same day. The Parsortix enriched cells were harvested and deposited onto a microscope glass slide through centrifugation at 190 × g for 7 min. Subsequently, the slides were air-dried overnight at room temperature and stored at -80 °C until immunocytochemical analysis.

### *Prostatic plexus* blood processing

A volume of 15 ml blood from the *prostatic plexus* was processed within two hours after collection. Due to blood clots and high viscosity, cell enrichment with neither CellSearch nor Parsortix System was possible. Instead, PBMCs were obtained through Ficoll-Paque™ density separation: the blood was transferred to a 50 ml tube and centrifuged at 400 × g for 10 min, the pellet was suspended in 30 ml 1 × PBS and layered with 20 ml Ficoll-Paque™, centrifuged at 400 × g for 30 min, and the interface and supernatant containing the mononuclear cells were transferred to a new 15 ml tube. The cells were washed with 9 ml 1 × PBS and 1 ml 1 × H-Lysis buffer (R&D systems, cat.no. WL1000) and centrifuged at 400 × g for 10 min. Finally, the pellet was resuspended in 900 µl RPMI 1640 medium and 100 µl DMSO for storage at -80 °C until subsequent analysis. The cryopreserved samples were thawed, resuspended in 10 ml RPMI 1640 medium and centrifuged at 300 × g for 3 min. The supernatant was discarded, and the cells were resuspended in 10 ml phosphate-buffered saline (PBS). Viable cell numbers were determined using the Vi-Cell XR cell counter and spun on microscopy slides with 400,000 cells per slide. Per patient, 1 slide was analyzed for CTCs as described below.

### Immunocytochemistry positive control

The prostate cancer derived LNCaP cell line (ATCC, cat.no. CRL-1740) was grown in RPMI 1640 medium (PAN biotech GmbH, cat.no. P04-17500). The medium was supplemented with 10% fetal bovine serum (Sigma Life Science, cat.no. F0804-500ML), 1% Glutamine (ThermoFisher Scientific, cat.no. J60573.A1), and 1% Streptomycin/Penicillin (ThermoFisher Scientific, cat.no. 15070063). The cells were cultivated in a humidity-controlled environment at 37 °C with 5% CO_2_ until reaching 70% confluency. Peripheral Blood Mononuclear Cells (PBMCs) from healthy, age-matched, male donors were obtained through density-based Ficoll-Paque™ (Merck, cat.no. GE17-1440–02) separation as before [17]. The PBMCs were mixed with LNCaP cells in a 100:1 ratio with a total of approximately 300,000 cells per cytospins to function as positive control for immunocytochemistry.

### Immunocytochemical detection of CTCs

Cytospins with cells after enrichment with Parsortix PC1 System or Ficoll density gradient were analyzed by microscopy after immunocytochemical staining to detect epithelial cells, using pan-keratin antibodies and DAPI as positive selection marker and CD45 as exclusion marker as before [17]. Slides were fixed with 0.5% paraformaldehyde (PFA; Carl Roth GmbH, cat.no. 0335.3) in phosphate buffered saline (PBS; Thermo Fisher Scientific, cat.no. 14190–094) for 10 min and underwent three 3-min washes with PBS. Blocking was carried out with 10% AB-Serum (Bio-Rad Medical Diagnostics GmbH, cat.no. 805135) in PBS for 10 min, followed by three 3-min washes with PBS. The cells were stained overnight at 4 °C using 4',6-diamidino-2-phenylindole (DAPI) at a dilution of 1:700 (Thermo Fisher Scientific, cat.no. D1306), pan-keratin antibody AE1/AE3 directly labeled with AF488 at a dilution of 1:700 (Thermo Fisher Scientific, cat.no. 53–9003-82), and CD45 directly labeled with AF647 at a dilution of 1:700 (BioLegend, cat.no. 368528). Finally, the cytospins were subjected to three 3-min washes with PBS and examined under a fluorescence microscope. Similar to the CellSearch System’s classification, cells enriched from the peripheral blood with the Parsortix PC1 System that were positive for DAPI and keratin but negative for CD45 were classified as CTCs. Epithelial cells identified in the *prostatic plexus* blood were classified as Keratin + /DAPI + /CD45- cells as these cells had not entered the circulation fully yet and this phenotype could potentially include both tumor cells and normal prostatic cells locally released during surgery.

### Single-cell, whole genome next generation sequencing

Single-cell, Whole Genome Amplification (WGA) was performed using the Ampli1 WGA Kit according to the manufacturer's instructions (Menarini Silicon Biosystems, cat.no WG001R) on Keratin + /DAPI + /CD45- cells identified in the peripheral blood Parsortix-enriched cell fraction and the *prostatic plexus* blood mononuclear cell fraction. The identified CTCs were transferred to a 200 µl tube by micromanipulation using glass capillaries with a bevel angle of 45° and an outer diameter of 40 µm, attached to the Transferman 4 m instrument (Eppendorf). The WGA products underwent purification using the QIAquickR PCR Purification Kit (28,104, Qiagen), and the DNA concentrations were measured using the Qubit 1X dsDNA HS Assay Kit (Q33231, Invitrogen). The quality of the amplified DNA was assessed through multiplex PCR targeting the housekeeping gene GADPH, following established protocols [18]. Samples that exhibited at least two detectable bands of 100 and 200 bp and a DNA concentration of at least 0.05 µg/µl were deemed to be of sufficient quality for subsequent sequencing. NGS was performed on the DNBseqTM platform at BGI Genomics. Copy number alterations (CNAs) were assessed using the Control-Freec analysis tool as described before [19]. Subsequently, the data were further analyzed and visualized using R, following the same methodology as described previously [18].

### Statistical analysis

The statistical significance between the number of CTC-positive (≥ 1 CTCs) and -negative (0 CTCs) patients was calculated using the Fisher’s Exact test, whereas the McNemar test was applied for paired analysis. The median CTC difference between independent patient groups and between paired data was assessed using the Wilcoxon rank sum test and the Wilcoxon signed rank test, respectively. Correlations between enrichment techniques and the resulting CTC-statuses and numbers of CTCs were assessed using Cohen’s kappa and Kendall’s tau, respectively. Survival analyses were performed using Kaplan-Meyer curves, log-rank test, and cox proportional hazard function. All analyses were performed on the online statistical platform In-Silico Online version 2.4.0 [20] and R version 4.4.0.

## Results

### CTCs in the peripheral blood after enrichment with the CellSearch and Parsortix systems

To investigate a potential surgery-related release of tumor cells in the blood circulation, peripheral blood was investigated for CTCs during prostatectomy. Early-stage PCa was histologically confirmed in 103 patients with 78% of patients having a combined Gleason score of 7. None of the patients received prior treatment and all underwent prostatectomy that took 3 h on average. Blood was collected within the first hour of surgery and within 2 h after surgery-end. For further analyses, the results of both blood samples were combined to lower the variability caused by the rarity and potential short half-life time of CTCs in the peripheral blood of early-stage prostate cancer patients [7, 21], to increase the likelihood of detecting a single CTC in context of the Poisson-distribution [22], but specifically to take the variability between surgeries into account [23]. Because CTCs can undergo a (partial) epithelial-mesenchymal transition (EMT) and thereby change their phenotype [12], two different CTC enrichment methods were applied and the results compared as before [24].

Using the CellSearch System, CTC quantification could be performed in peripheral blood from 101 out of 103 cases (Table 1). Out of these cases, 16 patients (16%) had 1 or more detectable CTCs in 7.5 ml peripheral blood during (*n* = 9) and/or after surgery (*n* = 9), with the maximum number of 3 CTCs. CTC quantification using the Parsortix System was successfully performed in peripheral blood form 98 out of 103 cases (Table 1). Out of these cases, 31 patients (32%) had 1 or more detectable CTCs in 7.5 ml peripheral blood during (*n* = 20) and/or directly after surgery (*n* = 15), with the maximum number of 8 CTCs.

**Table 1:** CTCs enrichment from peripheral blood

	**During surgery**	**Post-surgery**	**During and post-surgery combined**
CellSearch System (total)	90	91	101
CTC-positive cases	9	9	16
CTC median (pos. cases)	1	1	1
CTC range (pos. cases)	1—10	1—3	1—12
Parsortix System (total)	89	85	98
CTC positive cases	20	15	31
CTC median (pos. cases)	1.5	2	2
CTC range (pos. cases)	1—4	1—8	1—9
**Comparison Cell Search and Parsortix**			
Difference: median CTC number Wilcoxon test	*p =* 0.050	*p =* 0.064	*p =* 0.0067
Difference: CTC pos/neg cases McNemar test	*p =* 0.0088	*p =* 0.38	*p =* 0.0087
Correlation: CTC pos/neg cases	κ = 0.052	κ = -0.15	κ = -0.042
Cohen’s kappa	*p =* 0.57	*p =* 0.18	*p =* 0.55
Correlation: CTC number	τ = 0.056	τ = -0.146	τ = -0.047
Kendall’s tau	*p =* 0.60	*p =* 0.18	*p =* 0.54
**CellSearch and Parsortix combined**			
CellSearch + Parsortix (total)	99	99	101
CTC-positive cases	27	24	42
Median CTCs (pos. cases)	1	1	2
Range CTCs (pos. cases)	1—12	1—5	1—14

Statistical comparisons between the number of blood samples being CTC-positive/negative and the actual number of CTCs detected after enrichment with the CellSearch and the Parsortix systems. Results of both enrichment assays at both timepoints and their sum were compared

The median number of detectable CTCs was significantly higher after enrichment with the Parsortix System compared to the median number of detectable CTCs enriched with the CellSearch System (*p =* 0.0067, Wilcoxon test). Furthermore, significantly more blood samples were found to be CTC-positive (≥ 1 CTC) after Parsortix enrichment (*p =* 0.0087, McNemar test). Interestingly, no correlations between the number of CTC-positive cases (*p* > 0.05, Cohen’s kappa) and the number of identified CTCs (*p* > 0.05, Kendall’s tau) were detected in the paired samples after CTC enrichment with the two techniques. Because the CellSearch System enriches CTCs via EpCAM-based immunomagnetic capture, thus identifying cells with epithelial characteristic, and the Parsortix utilizes cell size and deformability allowing also for the enrichment of CTCs that may have undergone EMT or possess low/absent EpCAM expression, different cell populations could potentially be enriched. As such, both outcomes were combined to get a more comprehensive assessment of tumor dissemination. This combined approach helps overcome the limitations inherent in relying on a single enrichment strategy and aligns with emerging evidence that diverse CTC subpopulations may carry complementary prognostic and biological information [11, 19]. In total, CTC results of 101 patients were evaluable and 42 cases were found to have at least 1 or more CTCs, with a median number of 2 CTCs (range: 1–14; Table 1) in the CTC-positive cases only. Taken together, these results indicate that a combined usage of different CTC enrichment systems is important to capture as many CTCs as possible regardless of EpCAM expression or cell size and deformability.

**Fig. 1:**
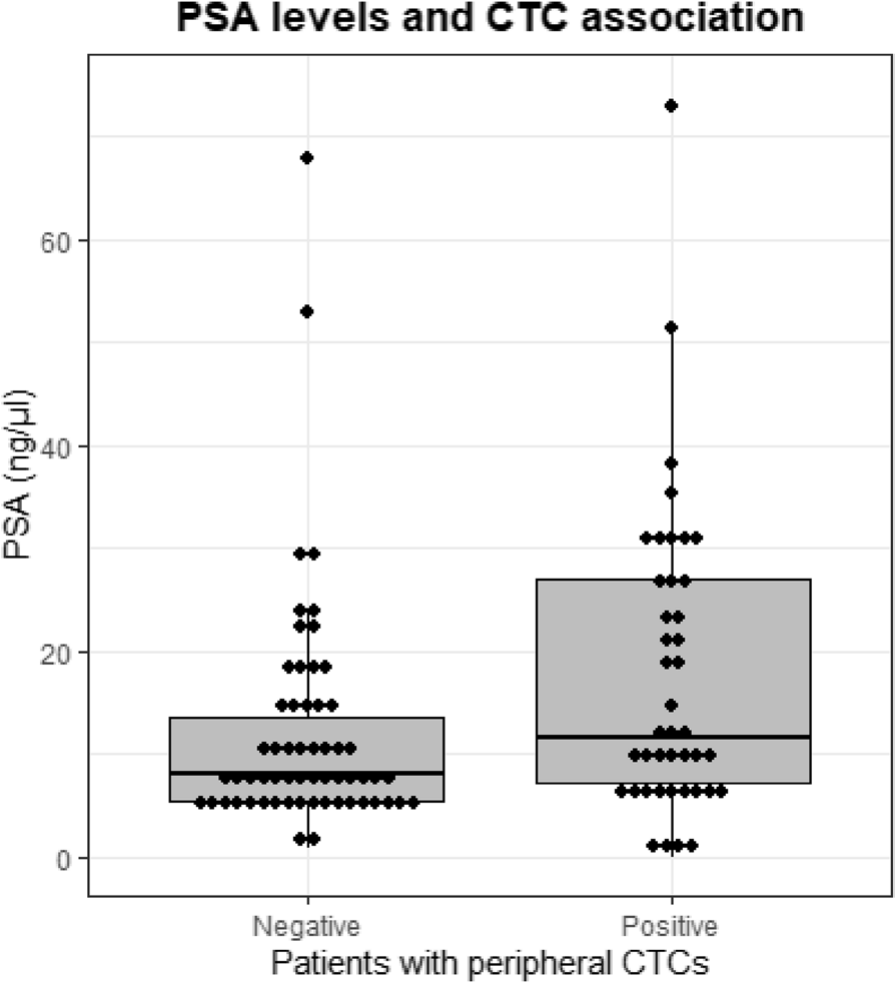
PSA levels and CTCs in peripheral blood. The presence of CTCs in the peripheral blood (x-axis) plotted versus the PSA levels (ng/μl) from the same patients (y-axis)

The presence of CTCs in the peripheral blood was significantly associated with the PSA levels at initial diagnosis; the median PSA was 3.5 ng/μl higher in CTC-positive cases compared to CTC-negative cases (*p =* 0.0094, Wilcoxon test; Table 2, Fig. 1). Additionally, the number of CTCs was positively correlated with these PSA levels (*r* = 0.24, *p =* 0.0153, Spearman’s rank correlation). No correlations were found with the CTCs and the clinical variables TNM-stages, Gleason score, and tumor volume. Furthermore, no association was detected between biochemical relapse (BCR) free survival and T-stage, N-stage, Gleason score, tumor volume, or CTCs (Supplementary Table 1). However, high PSA levels were associated with shorter BCR-free survival (*p* < 0.0001, Cox PH regression) as has been reported before [25], as well as PSA levels was positively correlated with tumor volume (*r* = 0.51, *p* < 0.0001, Pearson’s correlation).

**Table 2:** Number of cases identified with CTCs in peripheral blood after enrichment with the CellSearch System, Parsortix System, and both, correlated to clinical variables

	**CellSearch**			**Parsortix**			**CS + Px**		
**Tumor cell count**	**0**	**> 0**	***p*-val**	**0**	**> 0**	***p*-val**	**0**	**> 0**	***p*-val**
**T-stage**									
pT2c	29	4	0.7405	23	8	0.6021	21	12	0.7361
pT3a	35	7		28	13		24	18	
pT3b	21	5		16	10		14	12	
**N-stage**									
N0	66	12	0.7877	51	24	> 0.999	45	33	> 0.999
N1	18	4		15	7		13	9	
Nx	1	0		1	0		1	0	
**M-stage**									
M0	85	15	0.1584	67	30	0.3163	59	41	0.4158
M1	0	1		0	1		0	1	
**Gleason**									
7	66	12	0.7569	54	21	0.2669	47	31	0.5203
8	4	0		3	1		3	1	
9	15	4		10	9		9	10	
**Tumor volume**									
median (ml)	8.5	11.9	0.0690	8.7	10	0.3206	8.5	10	0.1375
**Pre-surgery PSA**									
median (ng/μl)	8.6	16.7	**0.0150**	8.4	12.5	**0.0334**	8.0	11.5	**0.0094**
**BRC-free survival**									
median (months)	39.1	NA	0.257	NA	NA	0.342	39.1	NA	0.114

### Tumor cells derived from the *prostatic plexus* blood during surgery

To investigate the potential surgery-related release of tumor cells in the blood at the site of surgery, blood from the *prostatic plexus* could be analyzed from 87 patients within the first hour of surgery and investigated for the presence of tumor cells. One or more tumor cells (Keratin + /DAPI + /CD45-) were detected in 74 (85.1%) patients with a median of 34 cells per 400,000 PBMCs. For easier comparison with the CTC measurements in peripheral blood, we extrapolated the number of detected tumor cells based on the number of PBMCs/ml to a total of 7.5 ml. As a result, the median number of tumor cells in 7.5 ml *prostatic plexus* blood was 97 (range: 0–6085).

**Fig. 2:**
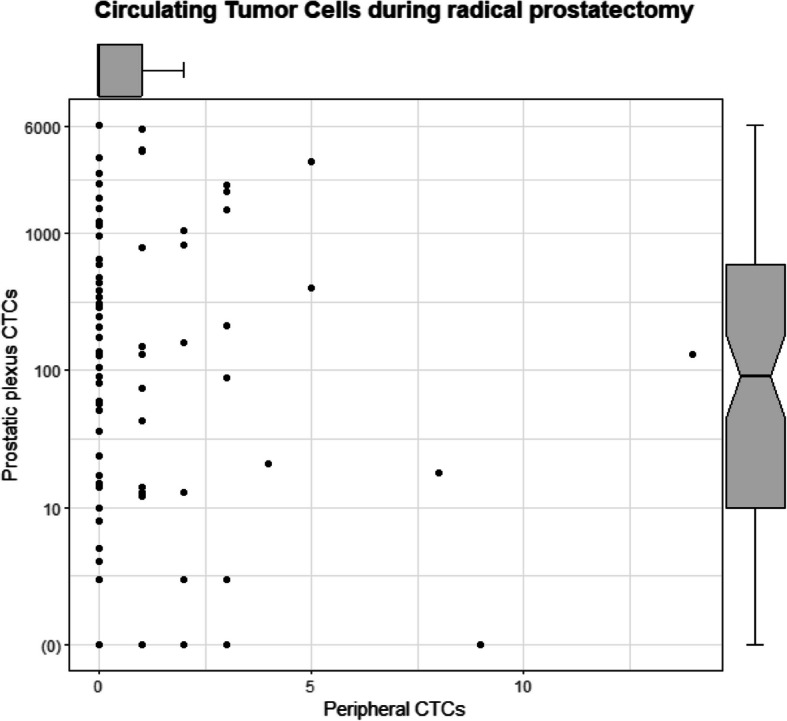
Distribution of detectable CTCs in different blood compartments. The number of CTCs identified in the *prostatic plexus* blood (y-axis, in log10-scale) plotted versus the CTCs from the same patients (*n* = 85) identified in peripheral blood during radical prostatectomy. Boxplots depict the median number of tumor cells. (0) = *prostatic plexus* CTC-negative

As can be seen in Fig. 2, compared to the median number of CTCs detected in the peripheral blood, the median number of detected tumor cells in the *prostatic plexus* blood was significantly higher (*p* < 0.0001, Wilcoxon test), as well as the number of cases with at least one detectable tumor cell (*p* < 0.0001, McNemar test). However, no correlation was found between the tumor cell presence (≥ 1 vs 0 cells) or their number and the blood of origin using one or both of the CTC enrichment techniques (*p* > 0.05, Kendall’s tau and Cohen’s kappa). Investigating the clinical variables, the number of patients with detectable tumor cells in the *prostatic plexus* blood was not correlated to Gleason score, TNM-stage, tumor volume, PSA, or BCR-free survival (Table 3).

**Table 3:** Number of cases identified with tumor cells in blood of *prostatic plexus* correlated with clinical variables

Tumor cell count	0	> 0	*p*-val	0–10	> 10	*p*-val	0–50	> 50	*p*-val	0–100	> 100	*p*-val	No clusters	Clusters	*p*-val
**T-stage**															
pT2c	4	25	0.8656	5	24	0.3462	10	19	0.7288	15	14	> 0.999	15	14	0.6831
pT3a	6	27		10	23		14	19		16	17		14	19	
pT3b	3	21		8	16		11	13		12	12		13	11	
**N-stage**															
N0	10	55	> 0.999	17	48	0.8366	26	39	0.8793	31	34	0.4444	33	32	0.8001
N1	3	17		6	14		9	11		12	8		9	11	
Nx	0	1		0	1		0	1		0	1		0	1	
**M-stage**															
M0	13	72	> 0.999	23	62	> 0.999	35	50	> 0.999	43	42	> 0.999	42	43	> 0.999
M1	0	1		0	1		0	1		0	1		0	1	
**Gleason**															
7	11	56	> 0.999	19	48	0.7984	29	38	0.7445	36	31	0.2642	35	32	0.2629
8	0	3		0	3		1	2		2	1		0	3	
9	2	14		4	12		5	11		5	11		7	9	
**Tumor volume**															
Median ml	8.5	9	0.6951	9	8.7	0.7474	9	8.7	0.8639	9	8.7	0.4841	9	8.5	0.8323
**PSA**															
median ng/ul	9.33	9.57	0.4584	7.03	9.68	0.1232	8.62	9.67	0.3699	9.33	9.47	0.6848	10.32	8.86	0.6377

Besides single tumor cells, tumor cell clusters were found in the blood from the *prostatic plexus* as well (Supplementary Fig. 1). Since CTC clusters have been shown to exhibit a higher metastatic potential than single CTCs in breast cancer and other solid tumors [17], we investigated the effect of the presence of CTC clusters on patient outcome. Tumor cell clusters were identified in 44/86 tumor cell positive cases (51.2%); however, the presence of cell clusters was not associated with biochemical relapse during the follow-up time of 25.2 months (*p =* 0.092, log-rank test).

Taken together, these results indicate that the tumor cells present in the *prostatic plexus* during surgery do not reflect the number or occurrence of CTCs in circulation, and their clinical relevance requires further investigation.

### Single-cell genomic analysis of epithelial cells from the peripheral blood and *prostatic plexus*

Because surgery may lead to mechanic dissociation of both normal and tumor cells, the origin of Keratin + /DAPI + /CD45- cells found in the blood of the *prostatic plexus* and peripheral blood was assessed by NGS. In total, we collected 26 cells from 12 different patients and analyzed their genome for copy number alterations (CNAs). Clear CNAs were identified in 13/19 cells from the *prostatic plexus* of 6 patients, as well as in 2/7 cells from peripheral blood of 7 patients (Supplementary Fig. 2).

**Fig. 3:**
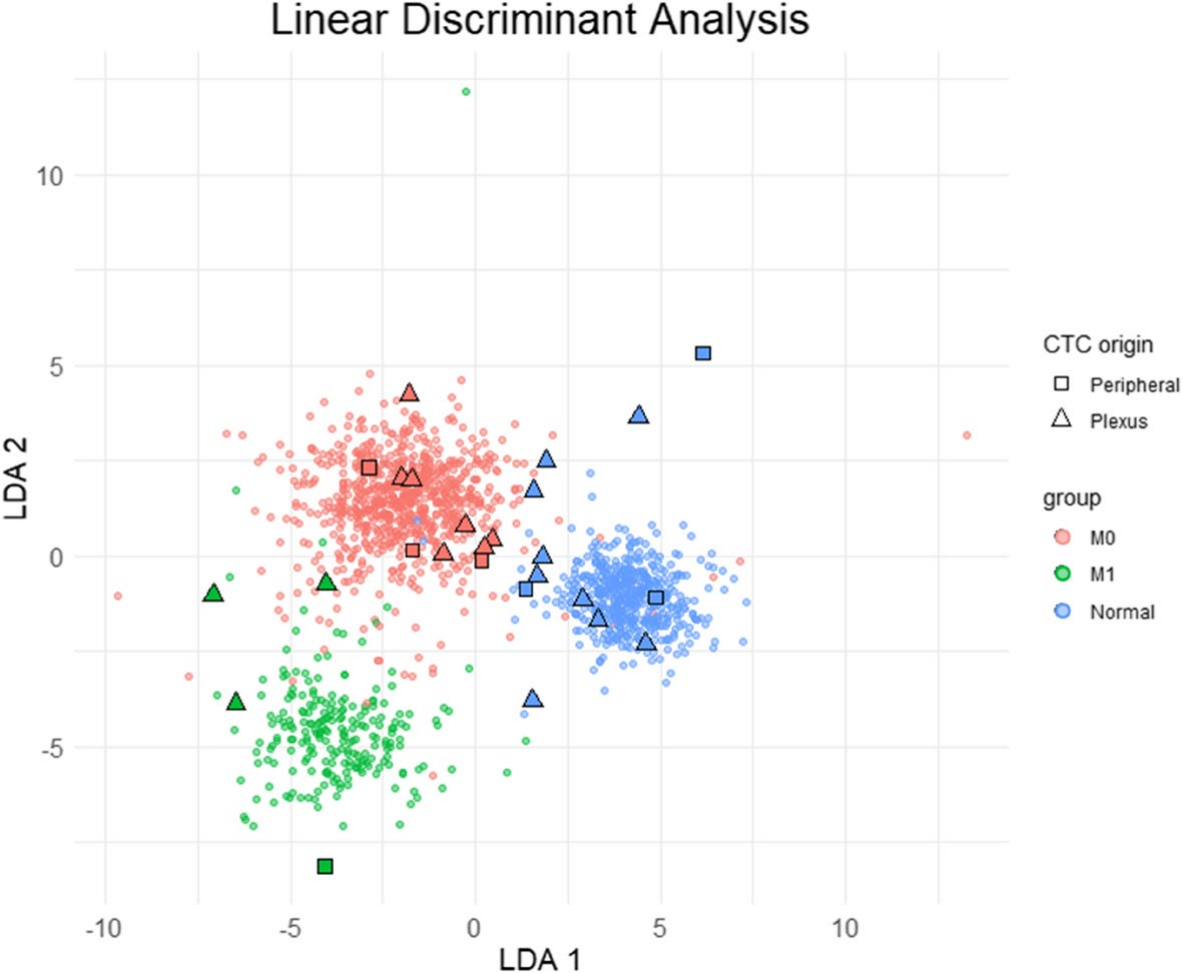
Classification of CTCs by Linear Discriminant Analysis (LDA). Copy number alteration (CNA) data from 1013 prostate cancers, including 787 early-stage (M0) and 226 advanced (M1) cases [Armenia et al., Nat Genet 2018], and 500 artificially generated profiles without CNAs (normal) were analyzed by LDA to classify 26 CTCs obtained from peripheral (squares) and *prostatic plexus* blood (triangles). The classification result of the CTCs is represented by the corresponding group colors

To classify whether these cells potentially exhibit metastatic potential, as well as to objectively discriminate epithelial cells in which no chromosomal aberrations were detected, a classifier was constructed to discriminate between early-stage prostate cancer, metastatic prostate cancer, and normal cells. CNA data originating from exome sequencing data from 1013 prostate cancer cases [26] were obtained through the Progenetix database (progenetix.org) [27] and 500 ‘normal’ profiles were generated with random noise based on our earlier NGS studies [18, 19]. The 1513 CNA profiles were used to create a Linear Discriminant Analysis (LDA) model, which was then used to classify the CNA profiles of the CTCs. Of the 19 (Keratin + /DAPI + /CD45-) cells extracted from the *prostatic plexus*, 10 were identified as tumor cells and 9 as normal epithelial cells; of the 7 (Keratin + /DAPI + /CD45-) cells from the peripheral blood, 4 were identified as tumor cells and 3 as normal epithelial cells (Fig. 3). Among the CTCs, 4 (3 from plexus blood and 1 from peripheral blood) were classified as most similar to metastatic prostate cancers (Supplementary Fig. 2). The distribution of tumor cells and normal epithelial cells between the prostatic plexus and peripheral blood among the randomly picked and investigated cells was similar (*p* > 0.99).

## Discussion

Local shedding of tumor cells into tumor-draining blood vessels has been reported for colorectal [28] and pancreatic cancer [29]. In the context of prostate cancer, we provided, for the first time, evidence that tumor resections can lead to considerable local release of epithelial cells (Keratin + /DAPI + /CD45-) into the blood of the *prostatic plexus*. Single cell DNA analysis of randomly selected CTCs indicates that at least part of these cells are tumor cells and some of these cells might even harbor a metastatic associated CNA signature.

Hematogenous dissemination is widely regarded as the main cause of distant metastasis in prostate cancer. However, the detection of CTCs in the peripheral blood of non-metastatic prostate cancer patients remains a challenge due to their phenotypic and genetic heterogeneity [9, 11, 30]. To address this issue, we enriched CTCs using two complementary FDA-cleared technologies: EpCAM-mediated capture (CellSearch System) and size-dependent capture (Parsortix System). By combining these approaches, we achieved higher detection rates than using either method alone. This result suggests that EpCAM-negative CTCs or small CTCs exists in early prostate cancer that can be missed by a single assay. In both systems, CTCs were identified by their expression of epithelial keratins and absence of the CD45 leukocyte antigen.

It is important to note that a complete loss of epithelial markers is rather rare in carcinomas and is usually linked to a lower metastatic potential [31, 32]. Nonetheless, we cannot rule out that some CTCs may have undergone EMT and downregulated these markers as has been shown before by others [33]. Moreover, by maximizing sensitivity through combining two complementary enrichment techniques, we inevitably captured a mixed cell population that may include both malignant and benign epithelial cells. Including additional (transcriptional) markers such PMSA or PSA, might increase specificity as recently described [34]. Despite these challenges, we verified the malignant nature of at least a subset of the identified epithelial cells with single-cell genomic copy number analysis. Their CNA profiles resembled those of metastatic and non-metastatic cancers that could serve as a risk estimator, however, further research and validation are needed. In some patients, we detected no CNAs in the captured Keratin + /DAPI + /CD45- cells from both the *prostatic plexus* and peripheral blood compartments, suggesting that these cells may instead be normal epithelial cells released during surgery. However, we cannot completely exclude the possibility that some epithelial cells with a normal copy number profile are in fact tumor cells, as discussed by the team of Scher and colleagues [35].

Parallel assessment of CTCs in the peripheral venous blood showed that there was no significant correlation between CTC detection in these two blood compartments. Besides technical reasons this might be explained by the dilution of CTCs in the peripheral blood and the assumption that surgically released tumor cells are rapidly cleared from the circulation by either shear stress or extravasation into a distant tissue such as bone marrow [36–39]. Therefore, it is imperative to conduct further research on the biology of tumor cells released during surgery to assess their viability and metastatic potential. Moreover, evaluation of clinical follow-up data over an extended period will be crucial to assess the long-term impact of surgery-related CTC release on disease progression and patient outcomes in PCa patients.

Taken together, our findings provide first insights into local release of tumor cells during prostatectomy and pave the road for future investigations of this important and under-investigated topic. Future studies may also include the side-by-side comparison of different surgical techniques or perioperative management. Drugs blocking the extravasation of CTCs into distant tissues are available [40–43] and one can imagine interventional trials testing the peri-operative use of these drugs to protect tumor cell spread similar to the use of antibiotics to prevent infections.

## Conclusion

This is the first study investigating the tumor draining vein during radical prostatectomy to assess local tumor cell release. Our results show a considerable local release of prostatic cells including cells with tumor-specific genomic aberrations. Thus, our study opens a new avenue for future studies on the molecular composition of locally released tumor cells as well as interventional trials assessing surgical techniques or extravasation-preventing drugs.

## Supplementary Information

Supplementary Material 1.

Supplementary Material 2.

## Data Availability

Data will be made available on reasonable request.
