# Supplementary material for: Comparative analysis of circulating tumor cells in prostatic plexus and peripheral blood of patients undergoing prostatectomy
Source: J Exp Clin Cancer Res. 2025 May 13;44:143. doi: 10.1186/s13046-025-03397-5 (PMC12070612; doi:10.1186/s13046-025-03397-5)
Supplement: Supplementary file 1 — Supplementary Material 1. [file 13046_2025_3397_MOESM1_ESM.docx]

**Supplementary Table 1 – Survival analysis.** Multivariable survival analysis (Cox proportional-hazards model) for biochemical relapse (BCR) and CTC status in peripheral blood, Gleason score, T-stage, N-stage, and pre-surgery PSA levels.

|  | **coef** | **exp(coef)** | **se(coef)** | **z** | **p** |
| --- | --- | --- | --- | --- | --- |
| CTC status (neg vs. pos) | 0.25943 | 1.29619 | 0.68157 | 0.381 | 0.7035 |
| Gleason score (7 vs. 8-9) | 0.73449 | 2.08443 | 0.47522 | 1.546 | 0.1222 |
| T-stage (T2 vs T3) | 0.05207 | 1.05345 | 0.48095 | 0.108 | 0.9138 |
| N-stage (N0 vs N1) | -0.29922 | 0.7414 | 0.55506 | -0.539 | 0.5898 |
| Tumor volume | -0.02187 | 0.97837 | 0.0338 | -0.647 | 0.5176 |
| Pre-surgery PSA levels | 0.08496 | 1.08868 | 0.01918 | 4.43 | 9.44E-06 |

Likelihood ratio test=20.96 on 6 df, p=0.003827

n= 92, number of events= 27
