# Supplementary material for: Comparative analysis of circulating tumor cells in prostatic plexus and peripheral blood of patients undergoing prostatectomy
Source: J Exp Clin Cancer Res. 2025 May 13;44:143. doi: 10.1186/s13046-025-03397-5 (PMC12070612; doi:10.1186/s13046-025-03397-5)

**Supplementary Figure 1** – Images of single (upper panels) and clusters (bottom panels) of CTCs detected in the *prostatic plexus* blood. Blue: DAPI, green: pan-keratin, red: CD45


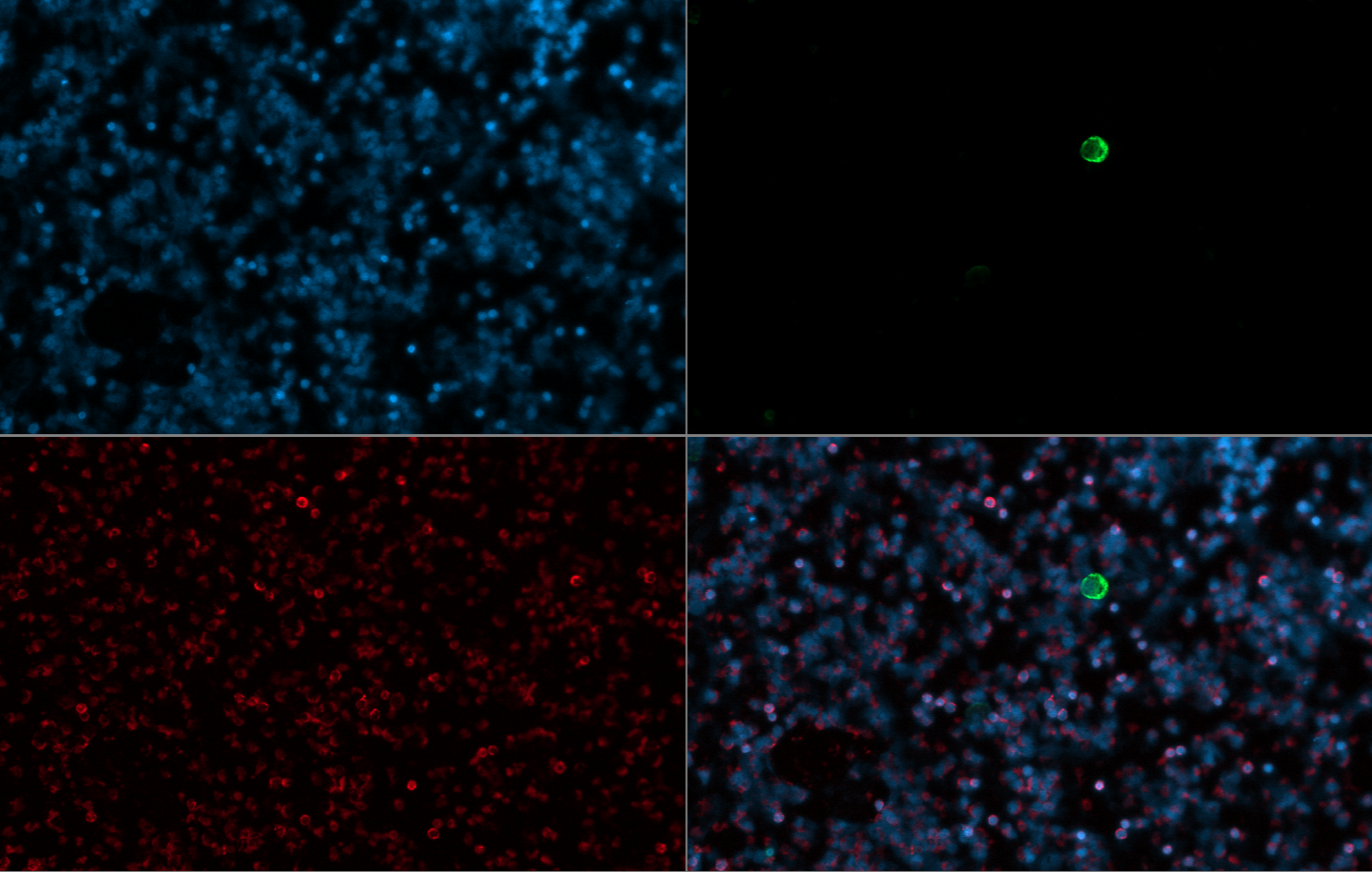


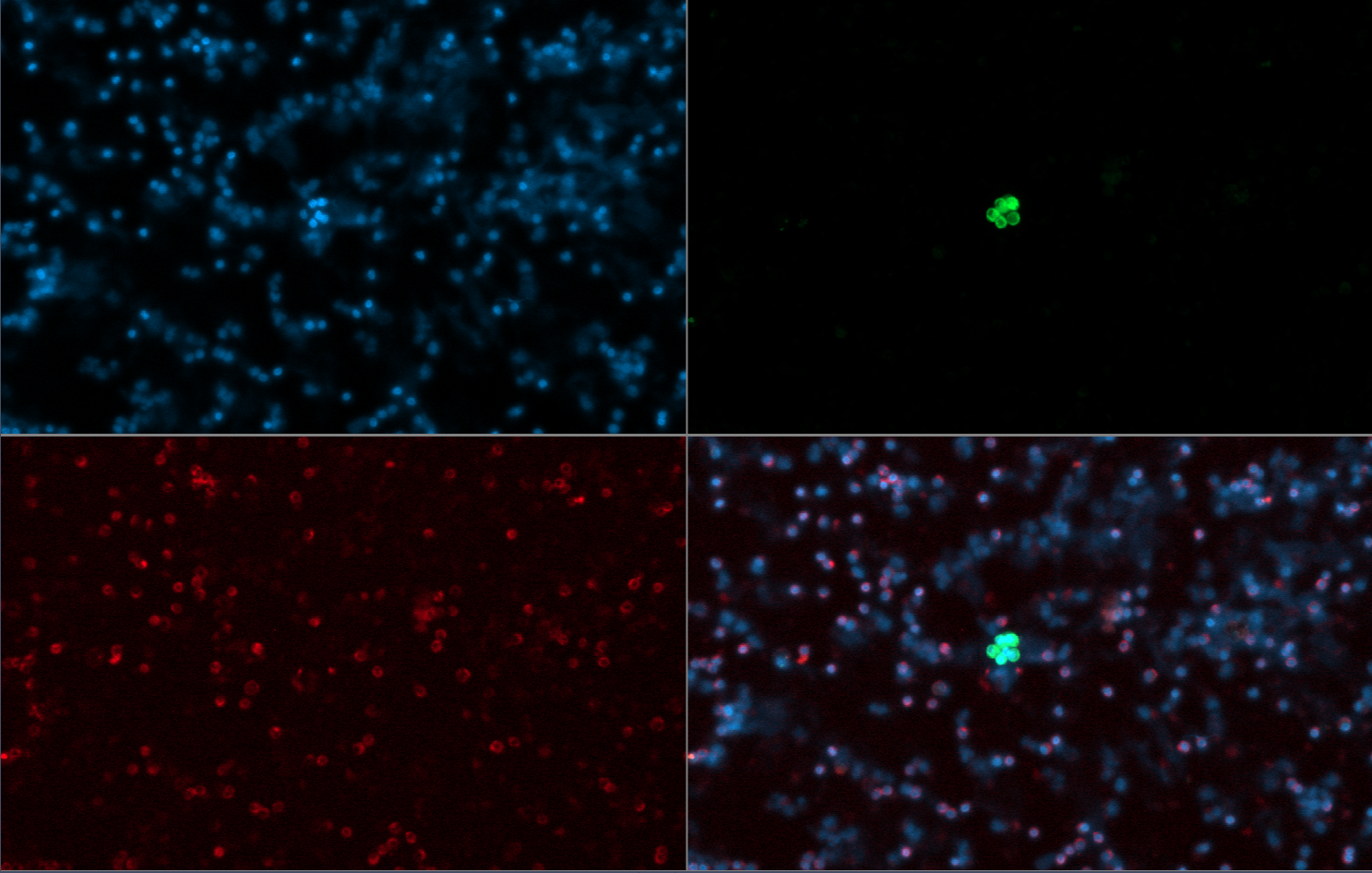


**Supplementary Figure 2** – Copy number profiles of single cells based in shallow, whole genome sequencing. Marked signals of log(ratio)>0 are considered copy number gain (green), whereas log(ratio)<0 are classified as losses (red). Values around a log(ratio) of 0 are normal (blue).


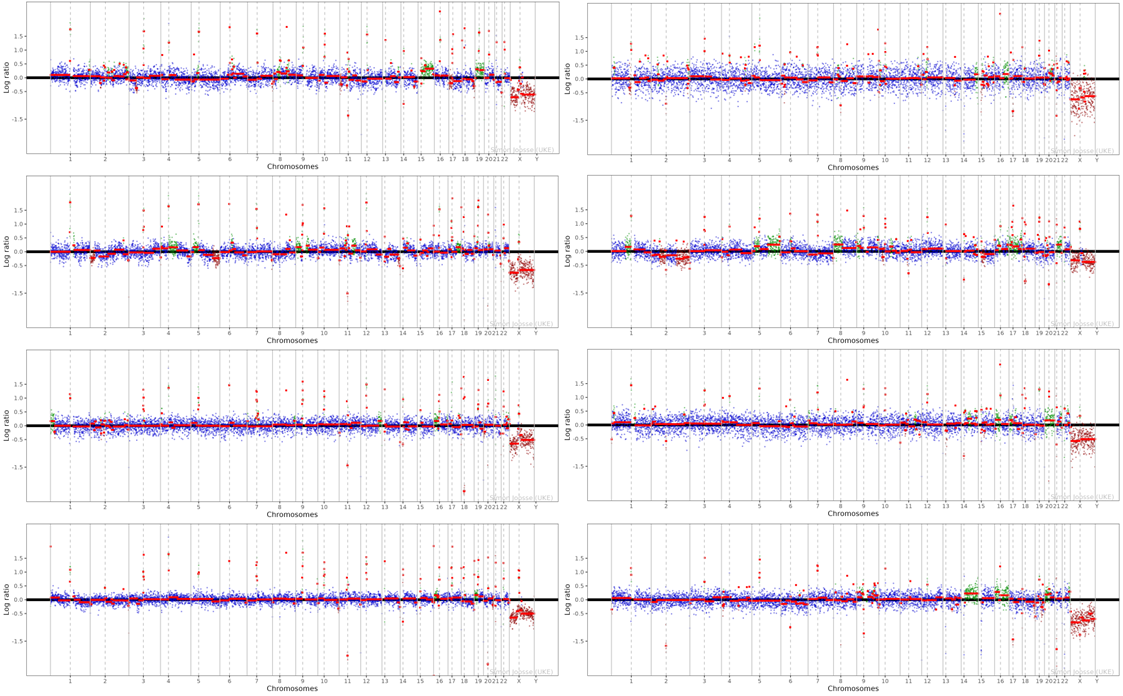

Supplement: Supplementary file 2 — Supplementary Material 2. [file 13046_2025_3397_MOESM2_ESM.docx]
